# A pharmacovigilance approach for assessing the occurrence of suicide-related events induced by antiepileptic drugs using the Japanese adverse drug event report database

**DOI:** 10.3389/fpsyt.2022.1091386

**Published:** 2023-01-09

**Authors:** Takenao Koseki, Mikako Horie, Satomi Kumazawa, Tetsuo Nakabayashi, Shigeki Yamada

**Affiliations:** ^1^Department of Clinical Pharmacy, Fujita Health University School of Medicine, Toyoake, Japan; ^2^Center for Regulatory Science, Pharmaceuticals and Medical Devices Agency, Tokyo, Japan

**Keywords:** antiepileptic drugs, suicidality, pharmacovigilance, Japanese adverse drug event report, perampanel hydrate

## Abstract

Increased suicidality after antiepileptic drug (AED) treatment remains controversial. This study aimed to investigate the occurrence of suicide-related events (SREs) in Japan. SREs signals with AEDs used orally were evaluated by calculating reporting odds ratios (RORs) and information components (ICs) using the Japanese Adverse Drug Event Report (JADER) database from April 2004 to December 2021. Additionally, factors affecting the occurrence of SREs and time-to-onset from the initial AED treatment were analyzed. Of 22 AEDs, 12 (perampanel hydrate, nitrazepam, levetiracetam, clonazepam, clobazam, sodium valproate, phenobarbital, lamotrigine, lacosamide, gabapentin, zonisamide, and carbamazepine) showed signals of SREs. Patients in their 20 and 30 s, female sex, and concomitant use of multiple AEDs affected the occurrence of SREs. In six AEDs, the median time-to-onset of SREs in patients taking all AEDs was <100 days. The pharmacovigilance approach revealed that several AEDs displayed suicidality signals. Female patients, those in their 20 and 30 s, undergoing combination therapy with ≥2 AEDs, and patients early (<100 days from the initial treatment) in the course of AED therapy should be cautioned about SREs.

## 1. Introduction

Pharmacotherapy with antiepileptic drugs (AEDs) is the main treatment to control epileptic seizures. Approximately 70% of people with epilepsy will achieve long-term remission from seizures with AEDs (1, 2). However, the risk of suicide during AED treatment remains controversial (3).

In 2008, based on a meta-analysis of 199 placebo-controlled randomized clinical trials, the Food and Drug Administration (FDA) issued a safety class label warning on the risk of suicidality associated with the following 11 AEDs: carbamazepine, divalproex, felbamate, gabapentin, lamotrigine, levetiracetam, oxcarbazepine, pregabalin, tiagabine, topiramate, and zonisamide (4). Subsequently, Klein et al. reported that there was no evidence of increased suicidality with five other AEDs (eslicarbazepine, perampanel, brivaracetam, cannabidiol, and cenobamate), which were approved by the FDA since 2008 (5). Other case-control and cohort studies have evaluated the relationship between AEDs use and suicidality in epilepsy. Increased risk for suicidality has been reported for several AEDs by some studies (6, 7), whereas no such increase was found by others (8–10). Therefore, results regarding the association between AEDs and suicidality risk are inconsistent, partly because of methodological limitations. Nearly all studies were case-controlled studies or meta-analyses; few used pharmacovigilance databases.

Recently, pharmacovigilance signal detection studies have been conducted using a large accumulated database of adverse events reported by a spontaneous reporting system (11–13). The Japanese Adverse Drug Event Report (JADER) is a nationwide database of spontaneous adverse reports published by the Pharmaceuticals and Medical Devices Agency (PMDA), a pharmaceutical regulatory authority in Japan. The JADER database contains data of approximately 760,000 patients and 1,250,000 adverse events reported after April 2004. It is useful for detecting signals of rare adverse events, such as suicide-related events (SREs) in patients receiving AEDs.

The present study investigated the signals of SREs, factors affecting the occurrence of SREs, and the time to onset of SREs in patients taking orally administered AEDs using the JADER database.

## 2. Materials and methods

### 2.1. Data source

**FIGURE 1:**
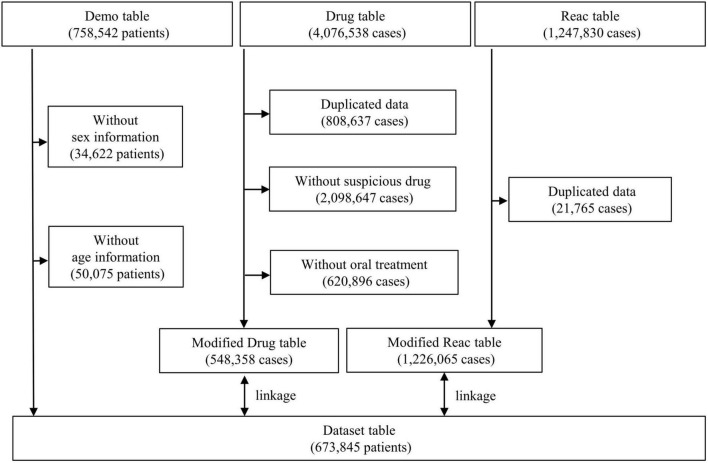
Flow diagram of the study.

Data from the JADER database (open-access database) between April 2004 and December 2021 were obtained from the PMDA website.^1^ The JADER dataset used in this study consisted of three data tables: demographic information “demo” table, drug information “drug” table, and adverse events information “reac” table, which included 758,542 patients, 4,076,538 cases, and 1,247,830 cases, respectively. The “demo” table included patient demographic data, such as sex and age. Patients with blank/unknown sex or age data in the “demo” table and those with duplicated data in the “drug” and “reac” tables were excluded. The demo table was linked to the “drug” and “reac” tables using the patient identification number of each case. In the “drug” table, the contribution of the drugs to the adverse events was classified into three categories: suspected drug, concomitant drug, and interaction. The “suspected drug” category was extracted in the present study. To evaluate the signals for SREs in patients who received AEDs orally, assuming they were outpatients, the AEDs with “oral” route of administration were selected. Data from 673,845 patients were included in this study (Figure 1).

### 2.2. Targeted antiepileptic drugs

Twenty-two orally administered AEDs approved for use in Japan (acetazolamide, acetylpheneturide, carbamazepine, clonazepam, clobazam, ethosuximide, ethotoin, gabapentin, lacosamide, lamotrigine, levetiracetam, nitrazepam, perampanel hydrate, phenytoin, phenytoin ⋅ phenobarbital, phenobarbital, primidone, sodium valproate, sultiame, topiramate, trimethadione, and zonisamide) were evaluated.

### 2.3. Definition of suicide-related events

Suicide-related events were extracted from the “reac” table according to the preferred terms (PTs) in the Medical Dictionary for Regulatory Activities (MedDRA 25.0 J). Fourteen PTs were determined from Suicide/self-injury (code 20000037) in the Standardized MedDRA Queries (SMQ), which are groups of PTs related to SREs (Table 1).

**TABLE 1:** Definition of suicide-related events.

SMQ code	SMQ name
20000037	Suicide/self-injury
PT code	PT name
10079105	Assisted suicide
10075616	Columbia suicide severity rating scale abnormal
10010144	Completed suicide
10012397	Depression suicidal
10022523	Intentional overdose
10022524	Intentional self-injury
10036000	Poisoning deliberate
10051154	Self-injurious ideation
10065604	Suicidal behavior
10042458	Suicidal ideation
10042464	Suicide attempt
10077417	Suicide threat
10082458	Suspected suicide
10081704	Suspected suicide attempt

PT, preferred term; SMQ, standardized MedDRA queries.

### 2.4. Signal detection

Because the pharmacovigilance database is based on reports of drug-induced adverse events and the population of patients taking AEDs is unknown, it is not possible to calculate the incidence of SREs occurred among patients taking AEDs. However, as the World Health Organization, PMDA, and other regulatory authorities have suggested, it is possible to estimate the potential risk of adverse events associated with a target drug by calculating parameters such as reporting odds ratios (RORs) and information components (ICs) based on two-by-two contingency tables (12). In this study, RORs and ICs were used for the signal detection of SREs as previously reported (14, 15). RORs, ICs, and their 95% confidence intervals (CIs) were calculated using a two-by-two contingency table (Table 2) and equations as described below. The calculations were performed using Excel for Microsoft 365 (Microsoft Corporation). The signals for SREs were positive when the lower limit of the 95% CI of the ROR exceeded 1, and that of the IC exceeded 0.

**TABLE 2:** Two-by-two contingency table.

	Target AEs	Other AEs	Total
Target drugs	*N*_11_	*N*_10_	*N_1+_*
Other drugs	*N*_01_	*N*_00_	*N_0+_*
Total	*N_+1_*	*N_+0_*	*N*_++_

AEs, adverse events; N, number of patients.

ROR equations:


$$\text{ROR}=\frac{N_{11}/N_{01}}{N_{10}/N_{00}}=\frac{N_{11}\,N_{00}}{N_{10}\,N_{01}}$$

$$\text{ROR}\,\left(95\%\,\text{CI}\right)=e^{\text{ln}\,\left(\text{ROR}\right)=1.96\,\sqrt{\frac{1}{N_{11}}+\frac{1}{N_{10}}+\frac{1}{N_{01}}+\frac{1}{N_{00}}}}$$

IC Equations:


$$E\,\left({\text{IC}}_{11}\right)=\log_{2}\frac{\left(N_{11}+\gamma_{11}\right)\,\left(N_{++}+\alpha \right)\,\left(N_{++}+\beta \right)}{\left(N_{++}+\gamma \right)\,\left(N_{+1}+\alpha_{1}\right)\,\left(N_{+1}+\beta_{1}\right)}$$

$$V\left({\text{IC}}_{11}\right)={\left(\frac{1}{\mathrm{ln2}}\right)}^{2}[\frac{N_{++}-N_{11}+\gamma -\gamma_{11}}{\left(N_{11}+\gamma_{11}\right)\,\left(1+N_{++}+\gamma \right)}+$$

$$\frac{N_{++}-N_{+1}+\alpha -\alpha_{1}}{\left(N_{1+}+\alpha_{1}\right)\,\left(1+N_{++}+\alpha \right)}+\frac{N_{++}-N_{+1}+\beta -\beta_{1}}{\left(N_{+1}+\beta_{1}\right)\,\left(1+N_{++}+\beta \right)}]$$

$$\gamma =\gamma_{11}\,\frac{\left(N_{++}+\alpha \right)\,\left(N_{++}+\beta \right)}{\left(N_{+1}+\alpha_{1}\right)\,\left(N_{+1}+\beta_{1}\right)}$$

$$\gamma_{11}=1,\alpha_{1}=\beta_{1}=1,\alpha =\beta =2$$

$$\text{IC}\,\left(95\%\,\text{CI}\right)=E\,\left({\text{IC}}_{11}\right)=2\,\sqrt{V\,\left({\text{IC}}_{11}\right)}$$

### 2.5. Factor analysis

To investigate the factors affecting the occurrence of SREs, univariable and/or multivariable logistic regression analyses were performed in patients with or without AEDs. Variables showing *p* < 0.1 in the univariable logistic regression analysis were entered in the multivariable logistic regression model. *P*-values < 0.05 were considered statistically significant. Statistical analysis was performed using EZR (Saitama Medical Center, Jichi Medical University, Saitama, Japan) as a graphical user interface for R version 4.0.3 (The R Foundation for Statistical Computing, Vienna, Austria). EZR is a modified version of R Commander (version 1.54), designed to add statistical functions frequently used in biostatistics (16).

### 2.6. Time-to-onset analysis

Time-to-onset analysis was performed using the periods from the day of the initial administration of AEDs in “drug” table to the day of the first occurrence of the SREs recorded in the “reac” table. Patients with missing values or those without data were excluded. The median period and interquartile range (IQR) and Weibull shape parameters (WSPs) were determined (17–19). WSPs consist of parameters α and β, which determine the scale and shape of the distribution function, respectively. A larger and smaller α indicates a wider and shrinking data distribution, respectively. The shape parameter β indicates a hazard without a reference population. The hazard considerations are as follows: 95% CI of β includes 1 (hazard constant over time; random failure type), lower limit of the 95% CI of β > 1 (hazard increases over time; wear-out failure type), and upper limit of the 95% CI of β < 1 (hazard decreases over time; initial failure type). We evaluated AEDs with >10 patients reporting SREs between the day of the initial administration and the day of the first occurrence of SREs. Statistical analyses were performed using JMP 13.0 (SAS Institute Inc., Cary, NC, USA).

### 2.7. Ethical approval

Ethics approval and consent to participate were not required since this study was performed using an open access database.

## 3. Results

### 3.1. Patient characteristics

Table 3 summarizes the characteristics of 673,845 patients included in the study. For all adverse events, there were more male patients and those in their 70 s. For SREs, there were more female patients and those in their 30 s. Among those with SREs, 389 patients received 1 ≤ AEDs; nearly all patients with 1 ≤ AED were female and in their 20 and 30 s. Among the targeted AEDs, no SREs were reported for these seven: acetazolamide, acetylpheneturide, ethosuximide, ethotoin, primidone, sultiame, and trimethadione.

**TABLE 3:** Sex- and age-specific patient population with antiepileptic drugs for suicide-related events.

		Sex		Age											
		Male	Female	<10 years	10 s	20 s	30 s	40 s	50 s	60 s	70 s	80 s	90 s	100 s	Total
	Patients with all AEs	344,184	329,661	24,639	19,646	24,680	39,063	54,773	85,837	149,705	173,259	89,186	12,795	262	673,845
	Patients with SREs	1,619	2,082	19	282	677	760	657	465	357	332	129	22	1	3,701
Patients with SREs using antiepileptic drugs	All antiepileptic drugs	150	239	4	36	92	98	59	44	31	18	7	0	0	389
	Levetiracetam	43	48	4	12	17	20	11	6	12	6	3	0	0	91
	Lamotrigine	27	46	0	5	19	22	13	10	3	1	0	0	0	73
	Sodium valproate	25	48	0	4	30	21	7	6	5	0	0	0	0	73
	Carbamazepine	27	40	0	11	14	12	11	11	4	2	2	0	0	67
	Clonazepam	9	23	0	1	8	9	7	3	1	3	0	0	0	32
	Nitrazepam	8	21	0	2	5	7	7	3	4	1	0	0	0	29
	Perampanel hydrate	14	14	0	2	5	11	1	7	1	1	0	0	0	28
	Zonisamide	9	8	0	2	2	1	3	1	2	4	2	0	0	17
	Phenobarbital	7	9	0	2	3	5	0	5	1	0	0	0	0	16
	Phenytoin	6	5	0	2	1	2	0	3	3	0	0	0	0	11
	Lacosamide	7	4	0	1	1	1	2	2	2	1	1	0	0	11
	Gabapentin	3	4	0	0	0	4	2	1	0	0	0	0	0	7
	Clobazam	2	4	0	1	1	2	1	1	0	0	0	0	0	6
	Topiramate	2	2	0	0	0	2	1	1	0	0	0	0	0	4
	Phenytoin ⋅ phenobarbital	0	1	0	0	0	1	0	0	0	0	0	0	0	1

AEs, adverse events; SREs, suicide-related events.

### 3.2. Suicide-related events signals

Table 4 shows RORs and CIs of AEDs for SREs. Signals were detected in patients who used 1 ≤ AEDs (all AEDs: ROR, 4.68 [95% CI 4.21–5.21] and IC, 2.06 [95% CI 1.91–2.22]). Among the AEDs with 1 ≤ SREs, signals for SREs were detected in 12: Perampanel hydrate, nitrazepam, levetiracetam, clonazepam, clobazam, sodium valproate, phenobarbital, lamotrigine, lacosamide, gabapentin, zonisamide, and carbamazepine. Of these, perampanel hydrate showed the highest signal index (ROR, 20.59 [95% CI 13.91–30.48] and IC, 3.52 [95% CI 2.96–4.09]); nitrazepam, levetiracetam, and clonazepam showed high signal indices (ROR, 12.47 [95% CI 8.55–18.20] and IC, 3.10 [95% CI 2.56–3.65]); (ROR, 8.95 [95% CI 7.23–11.97] and IC, 2.96 [95% CI 2.65–3.27]); (ROR, 8.73 [95% CI 6.11–12.46] and IC, 2.76 [95% CI 2.25–3.28], respectively). On the other hand, phenytoin ⋅ phenobarbital, topiramate, and phenytoin did not show SRE signals (ROR, 25.87 [95% CI 3.18–210.36] and IC, 0.93 [95% CI −1.33–3.19]); (ROR, 2.55 [95% CI 0.95–6.85] and IC, 0.95 [95% CI −0.35–2.25]); (ROR, 1.82 [95% CI 1.00–3.30] and IC, 0.76 [95% CI −0.08–1.60], respectively).

**TABLE 4:** Reporting odds ratios and information components of antiepileptic drugs for suicide-related events.

Antiepileptic drug	All AE patients	SRE patients	ROR	[95% CI]	IC	[95% CI]
All antiepileptic drugs	16,169	389	4.68	[4.21–5.21]	2.06	[1.91–2.22]
Perampanel hydrate	276	28	20.59	[13.91–30.48]	3.52	[2.96–4.09]
Nitrazepam	453	29	12.47	[8.55–18.20]	3.10	[2.56–3.65]
Levetiracetam	1,973	91	8.95	[7.23–11.97]	2.96	[2.65–3.27]
Clonazepam	701	32	8.73	[6.11–12.46]	2.76	[2.25–3.28]
Clobazam	231	6	4.83	[2.15–10.88]	1.62	[0.51–2.73]
Sodium valproate	2,906	73	4.74	[3.75–5.99]	2.12	[1.78–2.47]
Phenobarbital	641	16	4.65	[2.83–7.65]	1.91	[1.20–2.62]
Lamotrigine	3,199	73	4.29	[3.40–5.43]	1.99	[1.65–2.34]
Lacosamide	458	11	4.19	[2.30–7.63]	1.77	[0.92–2.61]
Gabapentin	336	7	3.86	[1.82–8.16]	1.49	[0.46–2.52]
Zonisamide	1,139	17	2.75	[1.70–4.45]	1.31	[0.62–2.00]
Carbamazepine	5,188	67	2.39	[1.88–3.05]	1.20	[0.85–1.56]
Phenytoin ⋅ Phenobarbital	8	1	25.87	[3.18–210.36]	0.93	[−1.33–3.19]
Topiramate	288	4	2.55	[0.95–6.85]	0.95	[−0.35–2.25]
Phenytoin	1,108	11	1.82	[1.00–3.30]	0.76	[−0.08–1.60]

AE, adverse events; CI, confidence intervals; IC, information component; ROR, reporting odds ratio; SRE, suicide-related events.

### 3.3. Factors associated with suicide-related events

To investigate the factors affecting the occurrence of SREs with AEDs, the effects of sex and age on the occurrence of SREs in all patients were first evaluated by multivariable analysis using logistic regression (Table 5). Since female outnumbered male patients among those with SREs and the 100 s was the center of the age groups in the percentage of SREs/all adverse events patients, male sex and 100 s age group were used as reference values. Multivariable analysis revealed that being female (odds ratio [OR] for females, 1.09 [95% CI 1.02–1.16], *p* = 0.013), and in ones’ 20 s (OR for 20 s, 7.43 [95% CI 1.04–53.00], *p* = 0.045) or 30 s (OR for 30 s, 5.23 [95% CI 0.73–37.30], *p* = 0.099) were associated with an increased occurrence of SREs.

**TABLE 5:** Odds ratios for suicide-related events.

	OR	95% CI	*P*-values
**Sex (vs. male)**			
Female	1.09	1.02–1.16	0.013
**Age (vs. 100 s)**			
<10 years	0.21	0.03–1.55	0.125
10 s	3.87	0.54–27.60	0.178
20 s	7.43	1.04–53.00	0.045
30 s	5.23	0.73–37.30	0.099
40 s	3.22	0.45–23.00	0.244
50 s	1.45	0.20–10.40	0.711
60 s	0.64	0.09–4.57	0.656
70 s	0.51	0.07–3.67	0.506
80 s	0.39	0.05–2.76	0.342
90 s	0.45	0.06–3.36	0.438

CI, confidence intervals; OR, odds ratio.

Second, we assessed the effect of sex (female), age (20 and 30 s), and the number of concomitant AEDs used (1 other AED or 2 ≤ other AEDs vs. AED monotherapy) on the occurrence of SREs in patients who used AEDs for which signals were detected (Table 6). Univariable analysis revealed that female sex and 20 and 30 s age groups, but not concomitant use of AEDs, were associated with an increased occurrence of SREs in patients who used 1 ≤ AEDs (all AEDs; OR for female sex, 1.54 [95% CI 1.25–1.89], *p* < 0.001; OR for 20 and 30 s age groups, 3.42 [95% CI 2.79–4.18], *p* < 0.001). SREs occurred more frequently in female patients treated with these four AEDs: nitrazepam, clonazepam, sodium valproate, and carbamazepine. SREs were more frequent among patients in their 20 and 30 s, with nearly all AEDs, excluding lacosamide and zonisamide. The occurrence of SREs tended to increase with concomitant use of one other AED in patients taking phenobarbital and carbamazepine and concomitant use of 2 ≤ other AEDs in patients taking perampanel hydrate, lamotrigine, and carbamazepine. Multivariable analysis confirmed almost all the results of the univariable analysis.

**TABLE 6:** Univariable and multivariable analysis for associated factors of suicide-related events with antiepileptic drugs.

	Univariable analysis			Multivariable analysis		
	OR	95% CI	*P*-values	OR	95% CI	*P*-values
**All antiepileptic drugs**						
Female sex	1.54	1.25–1.89	<0.001	1.35	1.09–1.66	0.005
20 and 30 s age groups	3.42	2.79–4.18	<0.001	3.29	2.69–4.04	<0.001
Concomitant use of 1 other antiepileptic drug	0.96	0.68–1.34	0.793			
Concomitant use of 2 ≤ other antiepileptic drugs	1.38	0.83–2.30	0.217			
**Perampanel hydrate**						
Female sex	1.38	0.63–3.03	0.415			
20 and 30 s age groups	4.47	2.00–9.99	<0.001	4.64	2.05–10.50	<0.001
Concomitant use of 1 other antiepileptic drug	1.99	0.73–5.45	0.179			
Concomitant use of 2 ≤ other antiepileptic drugs	2.58	0.93–7.17	0.069	2.49	0.89–7.00	0.084
**Nitrazepam**						
Female sex	4.75	2.05–11.00	<0.001	4.44	1.91–10.30	<0.001
20 and 30 s age groups	2.26	1.04–4.89	0.039	1.91	0.87–4.21	0.108
Concomitant use of 1 other antiepileptic drug	0.51	0.17–1.49	0.215			
Concomitant use of 2 ≤ other antiepileptic drugs	NA					
**Levetiracetam**						
Female sex	1.31	0.86–2.00	0.210			
20 and 30 s age groups	2.72	1.76–4.19	<0.001			
Concomitant use of 1 other antiepileptic drug	0.67	0.37–1.23	0.195			
Concomitant use of 2 ≤ other antiepileptic drugs	1.02	0.48–2.16	0.961			
**Clonazepam**						
Female sex	1.67	1.02–2.73	0.040	2.01	0.90–4.49	0.087
20 and 30 s age groups	2.77	1.69–4.55	<0.001	3.99	1.92–8.28	<0.001
Concomitant use of 1 other antiepileptic drug	0.60	0.24–1.49	0.269			
Concomitant use of 2 ≤ other antiepileptic drugs	0.73	0.21–2.49	0.615			
**Sodium valproate**						
Female sex	1.91	1.17–3.11	0.010	1.93	1.17–3.18	0.010
20 and 30 s age groups	8.04	4.84–13.40	<0.001	8.61	5.16–14.40	<0.001
Concomitant use of 1 other antiepileptic drug	0.48	0.25–0.91	0.026	0.37	0.19–0.72	0.003
Concomitant use of 2 ≤ other antiepileptic drugs	0.67	0.29–1.58	0.361			
**Phenobarbital**						
Female sex	1.68	0.62–4.57	0.310			
20 and 30 s age groups	3.84	1.42–10.40	0.008	3.71	1.36–10.10	0.010
Concomitant use of 1 other antiepileptic drug	2.80	0.84–9.32	0.093	1.80	0.64–5.08	0.268
Concomitant use of 2 ≤ other antiepileptic drugs	2.54	0.72–8.92	0.145			
**Lamotrigine**						
Female sex	0.97	0.60–1.57	0.896			
20 and 30 s age groups	2.28	1.43–3.63	<0.001	2.28	1.43–3.64	<0.001
Concomitant use of 1 other antiepileptic drug	0.44	0.16–1.23	0.117			
Concomitant use of 2 ≤ other antiepileptic drugs	2.07	0.88–4.88	0.096	2.21	0.95–5.28	0.066
**Lacosamide**						
Female sex	0.67	0.19–2.32	0.528			
20 and 30 s age groups	0.77	0.16–3.63	0.742			
Concomitant use of 1 other antiepileptic drug	2.29	0.56–9.35	0.249			
Concomitant use of 2 ≤ other antiepileptic drugs	3.97	0.77–20.5	0.100			
**Gabapentin**						
Female sex	1.57	0.35–7.13	0.558			
20 and 30 s age groups	6.79	1.48–31.20	0.014			
Concomitant use of 1 other antiepileptic drug	NA					
Concomitant use of 2 ≤ other antiepileptic drugs	NA					
**Zonisamide**						
Female sex	0.89	0.34–2.33	0.816			
20 and 30 s age groups	0.92	0.26–3.23	0.896			
Concomitant use of 1 other antiepileptic drug	1.84	0.66–5.12	0.241			
Concomitant use of 2 ≤ other antiepileptic drugs	0.49	0.06–3.88	0.501			
**Carbamazepine**						
Female sex	1.67	1.02–2.73	0.040	1.57	0.95–2.57	0.076
20 and 30 s age groups	2.77	1.69–4.55	<0.001	2.48	1.50–4.11	<0.001
Concomitant use of 1 other antiepileptic drug	1.89	1.00–3.58	0.051	1.76	0.93–3.34	0.083
Concomitant use of 2 ≤ other antiepileptic drugs	2.35	1.00–5.54	0.051	1.97	0.83–4.71	0.126

CI, confidence intervals; OR, odds ratio; NA, not applicable.

### 3.4. Time-to-onset of suicide-related events

**FIGURE 2:**
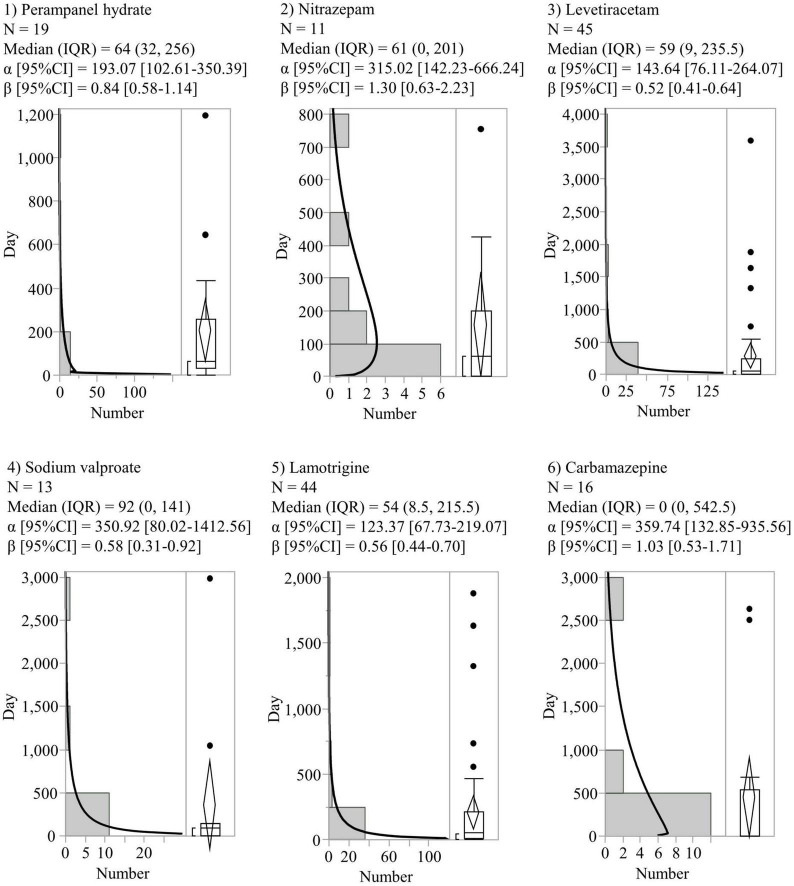
Histograms and Weibull shape parameters of suicide-related events. CI, confidence intervals; IQR, interquartile range; N, number of patients.

In the time-to-onset analysis, six AEDs (perampanel hydrate, nitrazepam, levetiracetam, sodium valproate, lamotrigine, and carbamazepine) with more than 10 reported SREs were evaluated. The histograms and WSPs of each AED are shown in Figure 2. The median period (IQR) to onset of SREs in patients with AEDs was as follows: perampanel hydrate (64 [32–256] days, *n* = 19), nitrazepam (61 [0–201] days, *n* = 11), levetiracetam (59 [9–235.5] days, *n* = 45), sodium valproate (92 [0–141] days, *n* = 13), lamotrigine (54 [8.5–215.5] days, *n* = 44), and carbamazepine (0 [0–542.5] days, *n* = 16). Levetiracetam, sodium valproate, and lamotrigine showed a lower limit of 95% CI of WSP β < 1 (initial failure type). No AEDs were reported with an upper limit of 95% CI of WSP β > 1.

## 4. Discussion

Suicide-related event signals, the factors affecting the occurrence of SREs, and their time-to-onset were studied based on the JADER database in Japan. Among the 22 targeted AEDs, the occurrence of one or more SREs was reported in 15 and SREs signals were detected in 12. SREs were more frequent among patients in their 20 and 30 s among patients across nearly all AEDs, in females, and with concomitant use of other AEDs in patients using some AEDs. In addition, some AEDs were classified as the initial failure types. Our results obtained using the pharmacovigilance approach may provide new insights into suicidality risk associated with AEDs.

Among the AEDs associated with SREs, perampanel hydrate, nitrazepam, levetiracetam, and clonazepam showed high SREs signal indices. Perampanel hydrate is an orally active, non-competitive, selective glutamate α-amino-3-hydroxy-5-methyl-4-isoxazolepropionic acid (AMPA) receptor antagonist (20). In a recent meta-analysis, perampanel hydrate did not show the risk of suicidal ideation and suicidal attempt in patients without a history of suicidality; and suicidal ideation (including suicide attempt) was reported in only 3 of 1119 (0.27%) patients treated with perampanel hydrate (5). Conversely, suicidal ideation, intentional drug overdose, and suicide attempt were reported in 10 of 482 (2.1%) patients treated with perampanel hydrate, who had no medical history of suicidal behavior in an observational 52-week cohort study (21); although a history of suicidal behavior could not be determined, our results suggest that patients with epilepsy should be monitored for signs of suicidal behavior during treatment with perampanel hydrate. The neurobiological mechanism of suicidality is still unknown. Upregulation of the binding of the AMPA receptors in the caudate nucleus of individuals who had completed suicide has been reported (22, 23). Antagonism of AMPA receptors by perampanel hydrate might be involved in suicidal behaviors, as indicated by the alteration of AMPA receptor function in patients who completed suicide. In addition, it has been reported that individuals taking perampanel hydrate have a high frequency of aggression, and that aggression is strongly correlated with suicidality risk (21, 24). Aggression is associated with increased glutamate levels in the amygdala, hypothalamus, and periaqueductal gray matter, and with stimulation of glutamate receptors (25, 26). Blockade of AMPA receptors can either increase or decrease aggressive behavior (27, 28). The *N*-methyl-D-aspartate (NMDA) receptor antagonist, phencyclidine, is associated with increased aggression at low doses, and reduced aggression at higher doses (29). Perampanel hydrate may cause increased aggression through AMPA receptor antagonism and subsequent alteration of NMDA receptor function, and the increased aggression may be associated with increased suicidality risk. Further, the frequency of NMDA-dependent spontaneous excitatory postsynaptic currents (EPSCs) is higher in the hippocampus of patients with mesial temporal lobe epilepsy than in non-epileptic controls (30), so the potential risk of SREs associated with perampanel hydrate use may be particularly high in patients with epilepsy. Benzodiazepines as antiepileptics, including nitrazepam and clonazepam were not included in the FDA’s meta-analysis; however, several case reports and case cohort studies have reported that these benzodiazepines increased showed suicidality risk or contributed to the cause of suicidality in various populations (6, 31–34). Our results support these reports and confirm that patients treated with benzodiazepines are at risk for suicidality. In general, benzodiazepines may reduce anxiety in epilepsy and lower suicidality risk owing to their gamma-aminobutyric acid (GABA) agonist properties (35). However, it was reported that cerebrospinal fluid concentrations of GABA were higher in individuals with a history of suicidal behavior than in those without this history (36), indicating that the effects of benzodiazepines on suicidality may depend on differences in individual neural abnormalities, including GABAergic neurons in patients with epilepsy. Several case-crossover/control studies reported that levetiracetam also increased the risk of suicidality (6, 7, 37, 38). Levetiracetam is thought to suppress seizures in patients with epilepsy by acting on synaptic vesicle protein 2A and AMPA receptors to decrease the amplitude and frequency of miniature EPSCs in cortical neurons (39, 40). As observed with perampanel hydrate, the AMPA receptor may play an important role in the occurrence of SREs in levetiracetam-treated patients.

In addition, being female, in ones’ 20 and 30 s, and concomitant use of AEDs increase the occurrence of SREs for some AEDs. Overall suicidality risk increases with age and is higher in men in the general population, whereas it decreases with age and is higher in female with epilepsy (41). Despite differences between patients with epilepsy and those receiving AEDs, we found that female patients, those in their 20 and 30 s, and who are on multiple AEDs are at higher risk for SREs, consistent with this report. Few studies have focused on the differential impact of monotherapy vs. combination therapy with one or more other concomitant AEDs on the suicidality risk in patients receiving AEDs. In our study, the risk of SREs was not affected by concomitant use of one or more other AEDs compared to AED monotherapy in patients receiving AEDs overall. Increased risk of SREs was seen in patients concomitantly using perampanel hydrate, lamotrigine, and carbamazepine with 1 ≤ other AEDs. Carbamazepine and lamotrigine are considered AEDs with anti-suicidal properties because they improve mood in epileptic patients *via* serotonergic mechanisms of action (42). Additionally, carbamazepine decreases the antiepileptic effects and adverse events of concomitant AEDs by inducing cytochrome P450 isoenzymes (1). Contrary to expectations, carbamazepine and lamotrigine appear to increase suicidality risk in monotherapy as well as in combination therapy with other AEDs. The principle of epilepsy treatment should be started as AED monotherapy; if unsuccessful after titrating to an optimal dose, combination therapy with other AEDs should be considered (1, 43). In addition to changes in efficacy and safety due to drug interactions, it may be necessary to note an increased suicidality risk for some AEDs, such as perampanel hydrate, lamotrigine, and carbamazepine, when multiple AEDs are used in combination therapy. In time-to-onset analysis, all of the 6 AEDs evaluated in this study had a median time to SREs onset of <100 days, and 3 AEDs were classified as “initial failure type” and other three AEDs were classified as “random failure type.” In FDA’s report, a higher risk of suicidal behavior or ideation was observed as early as 1 week after the first dose and persisted over time for at least 24 weeks. In support of this report, our results suggest that suicide risk induced by AEDs is more likely to occur early in the course of epilepsy therapy and requires particular attention within the first 100 days of treatment.

Our study had several limitations. Because spontaneous reporting systems, such as JADER are passive reporting systems, many biases, such as under-reporting, over-reporting, and confounding by comorbidities, exist. There is a strong association between epilepsy and psychiatric diseases (41). Although epileptic patients with or without a history of psychiatric diseases have a high risk of suicidality (41), and suicidal risk induced by AEDs in patients with epilepsy was reported to have the largest estimated OR compared to that of psychiatric patients by subgroup analysis (4), the influence of comorbidity of psychiatric diseases cannot be ruled out. Furthermore, the number of SREs reported for several AEDs is small. To avoid false-positive detection, we defined SRE signals as those with a significant difference in both the RORs and ICs. Despite these limitations, we believe that our approach using a pharmacovigilance database will contribute to the discussion on suicidality induced by AEDs and the factors affecting it.

In conclusion, based on a pharmacovigilance database several AEDs, such as perampanel hydrate, nitrazepam, levetiracetam, and clonazepam showed SREs signals. Female patients, those in the 20 and 30 s, and using multiple AEDs concomitantly increased the risk for SREs. New insights from our results may help in understanding the association between AEDs and suicidality, and aid clinicians and other medical staff to predict and prevent suicidality induced by AEDs.

## Data availability statement

Publicly available datasets were analyzed in this study. This data can be found here: https://www.pmda.go.jp/safety/info-services/drugs/adr-info/suspected-adr/0003.html.

## Author contributions

TK designed this study and performed the statistical analyses. TK and MH conducted the survey using the JADER database. TK, MH, SK, TN, and SY drafted the manuscript. All authors approved the final manuscript.

## Footnotes

1
https://www.pmda.go.jp/english/index.html
